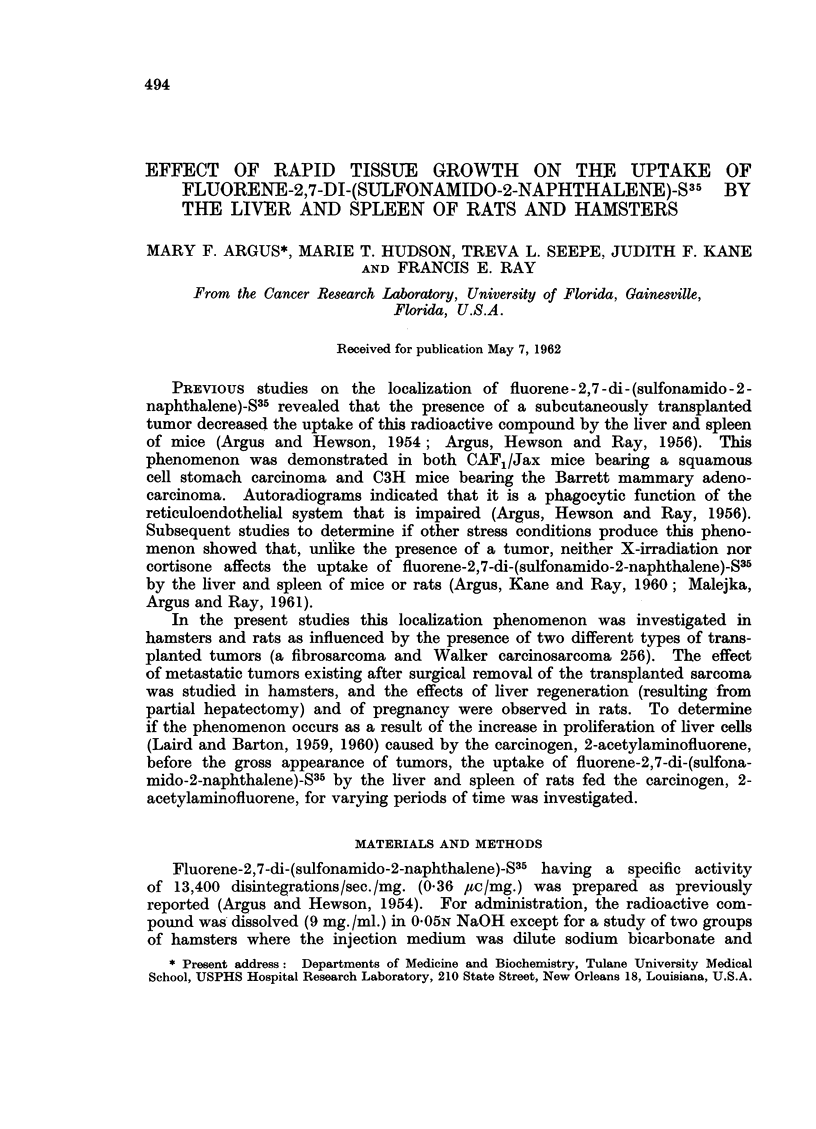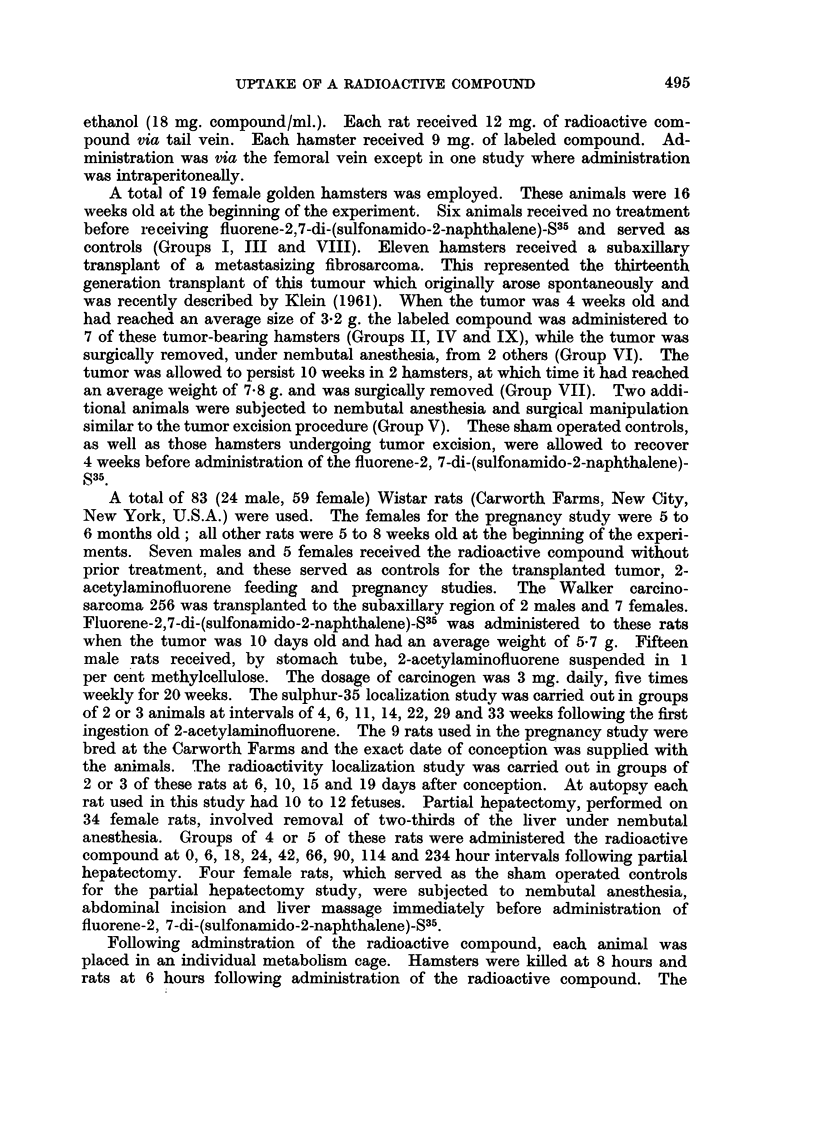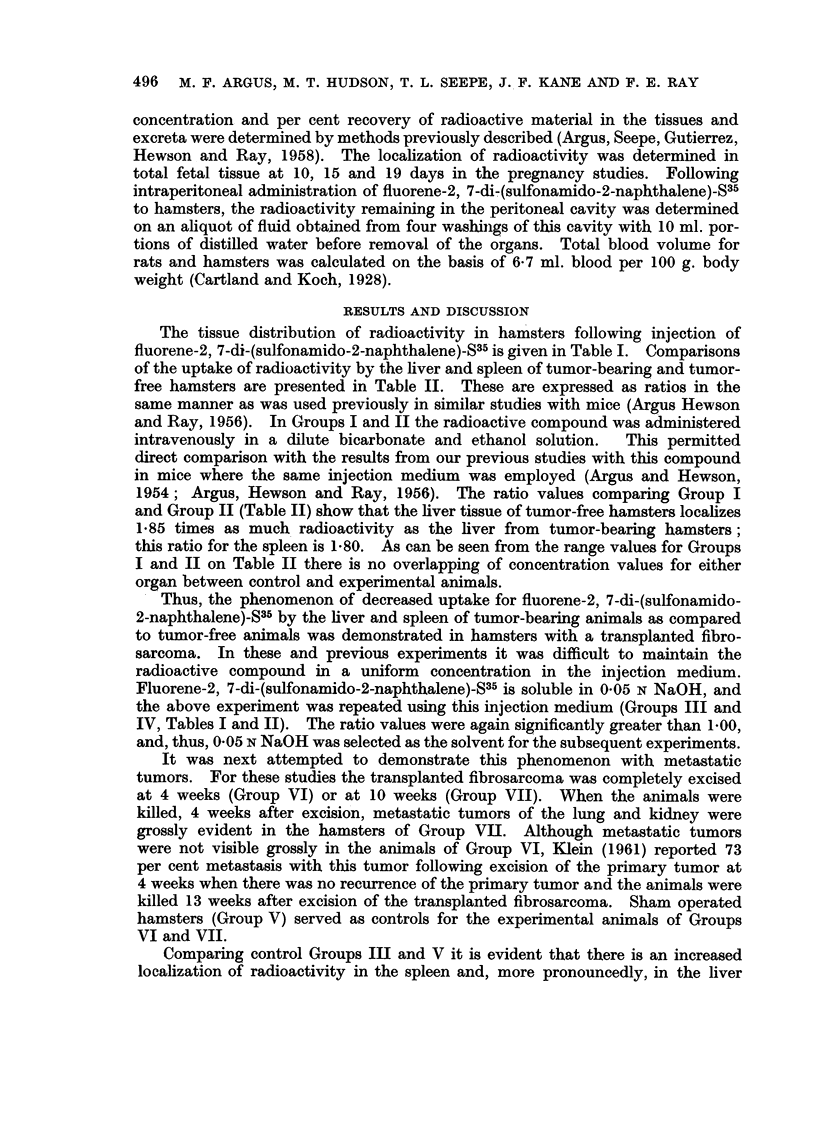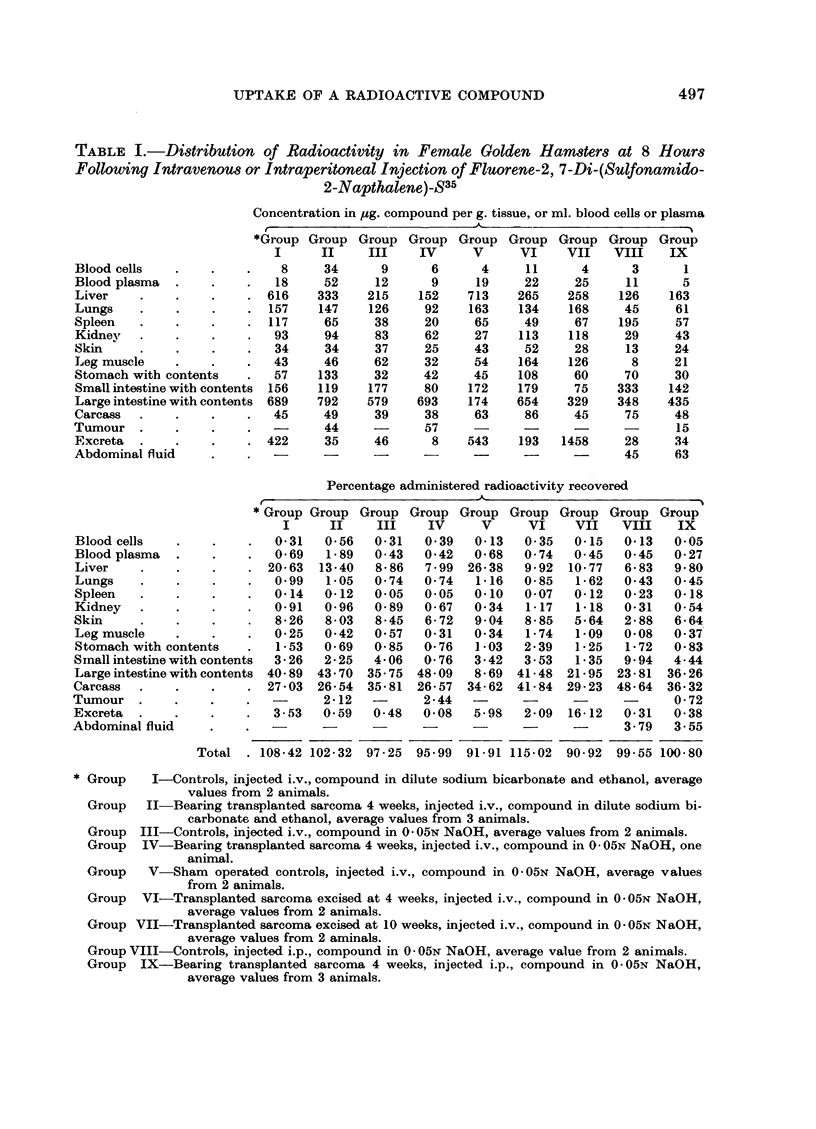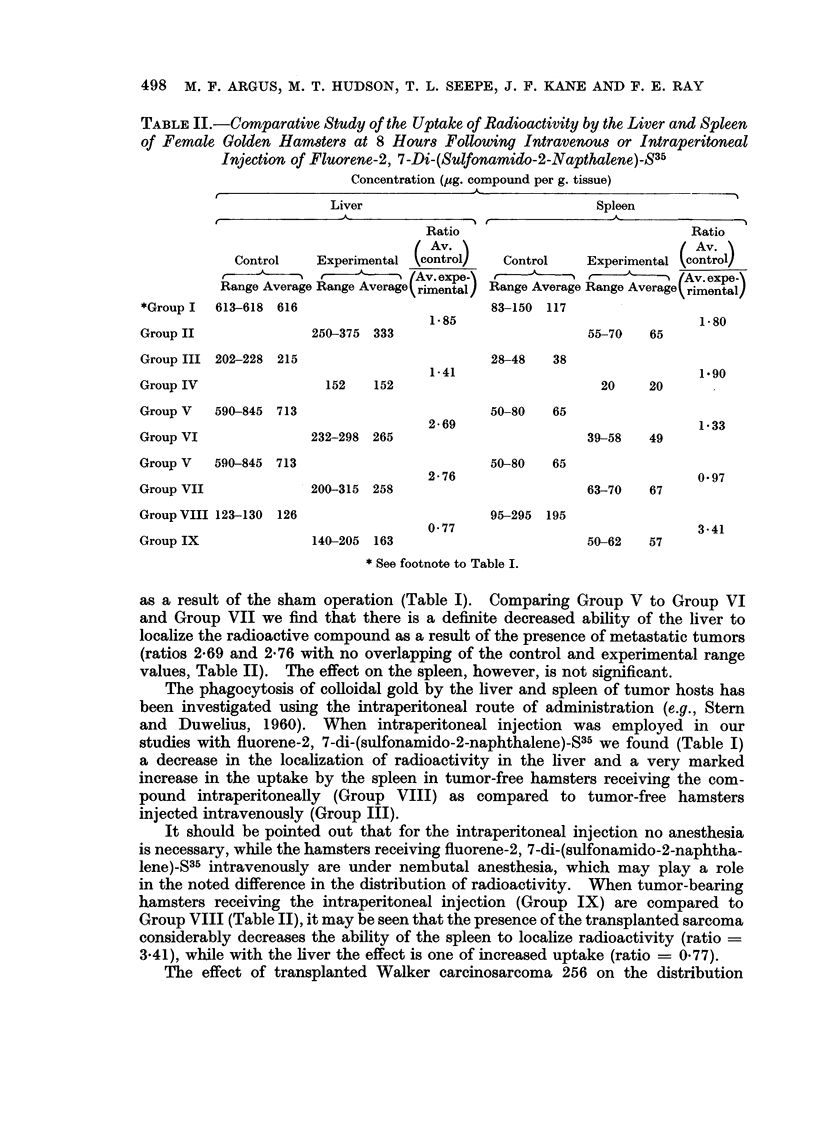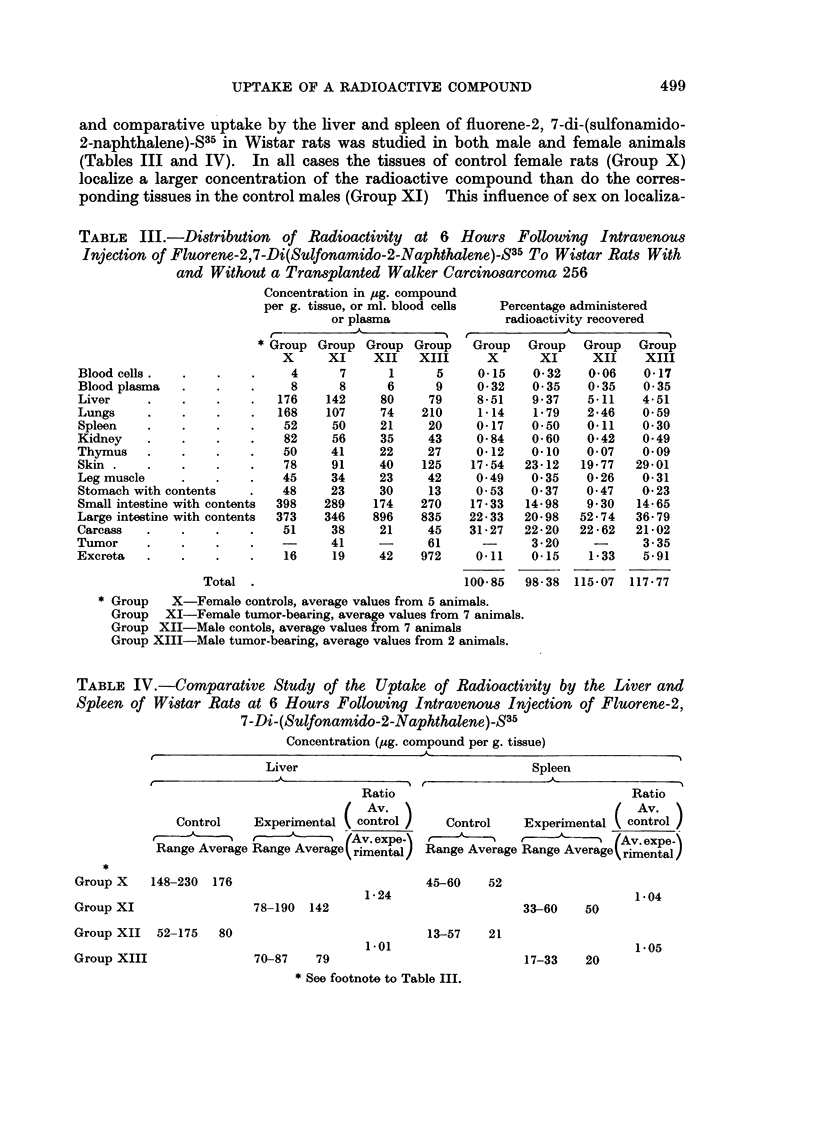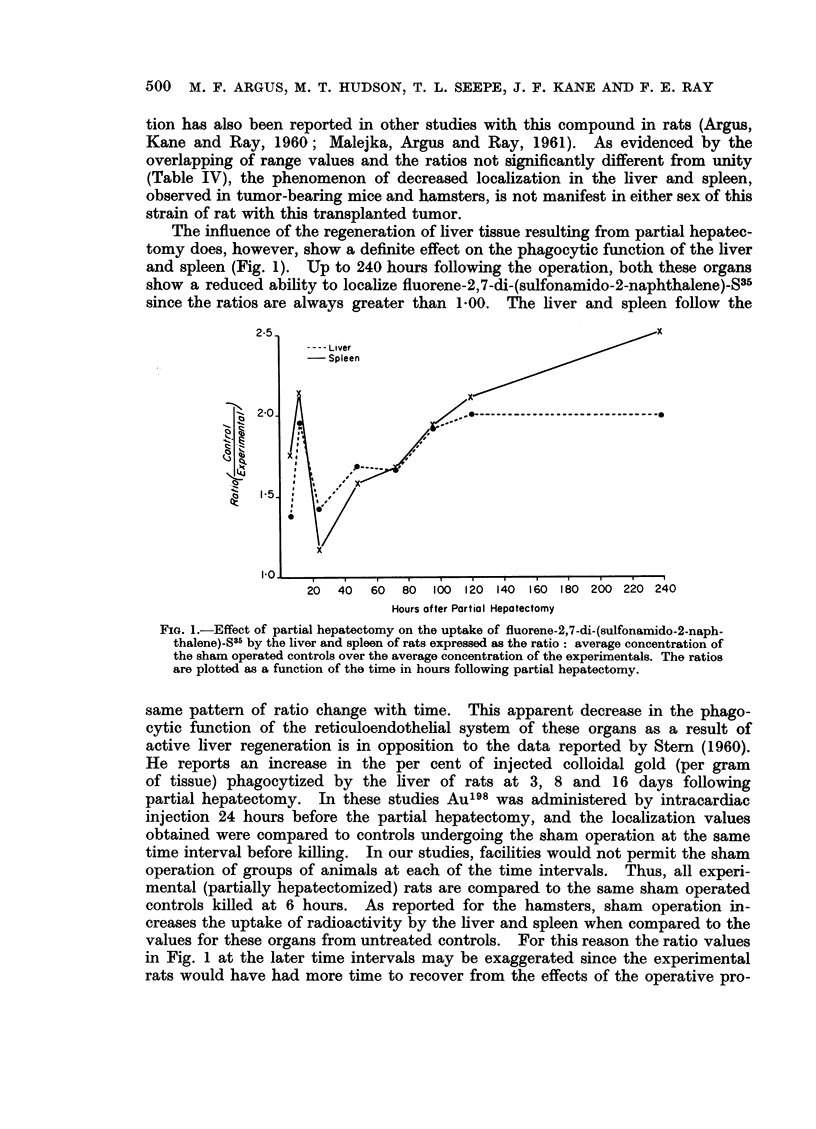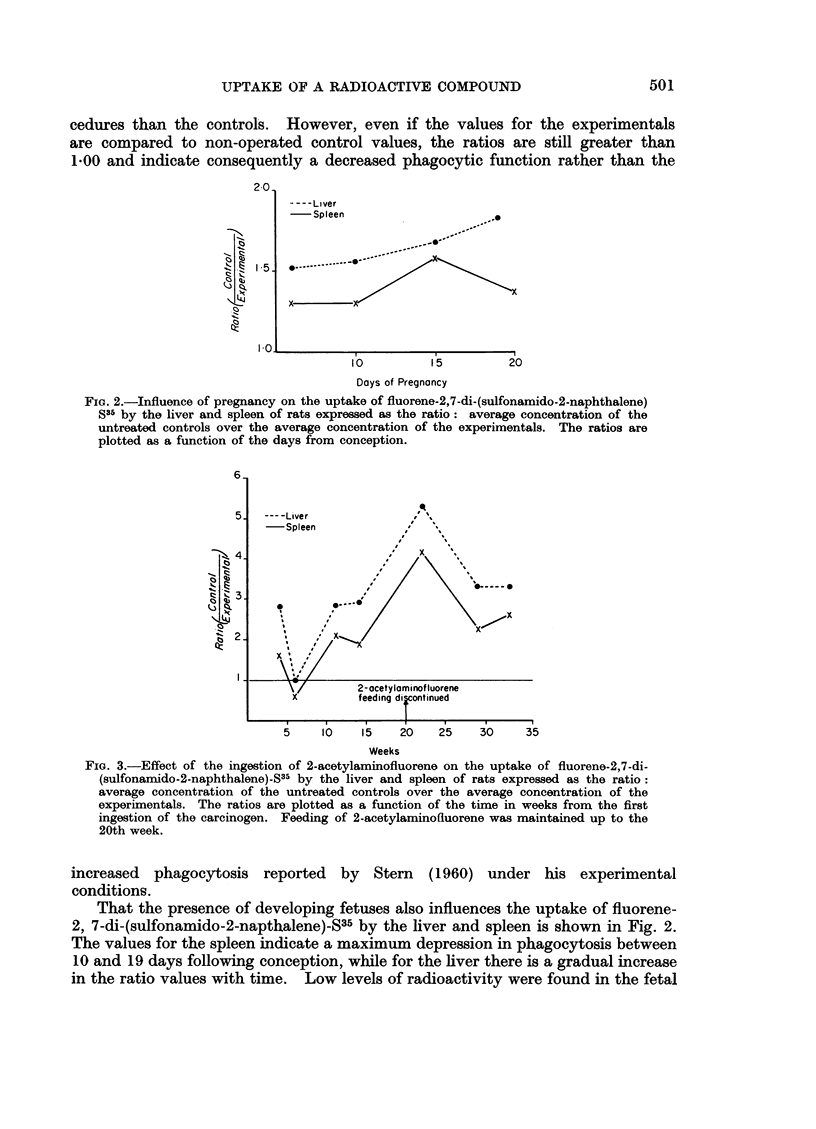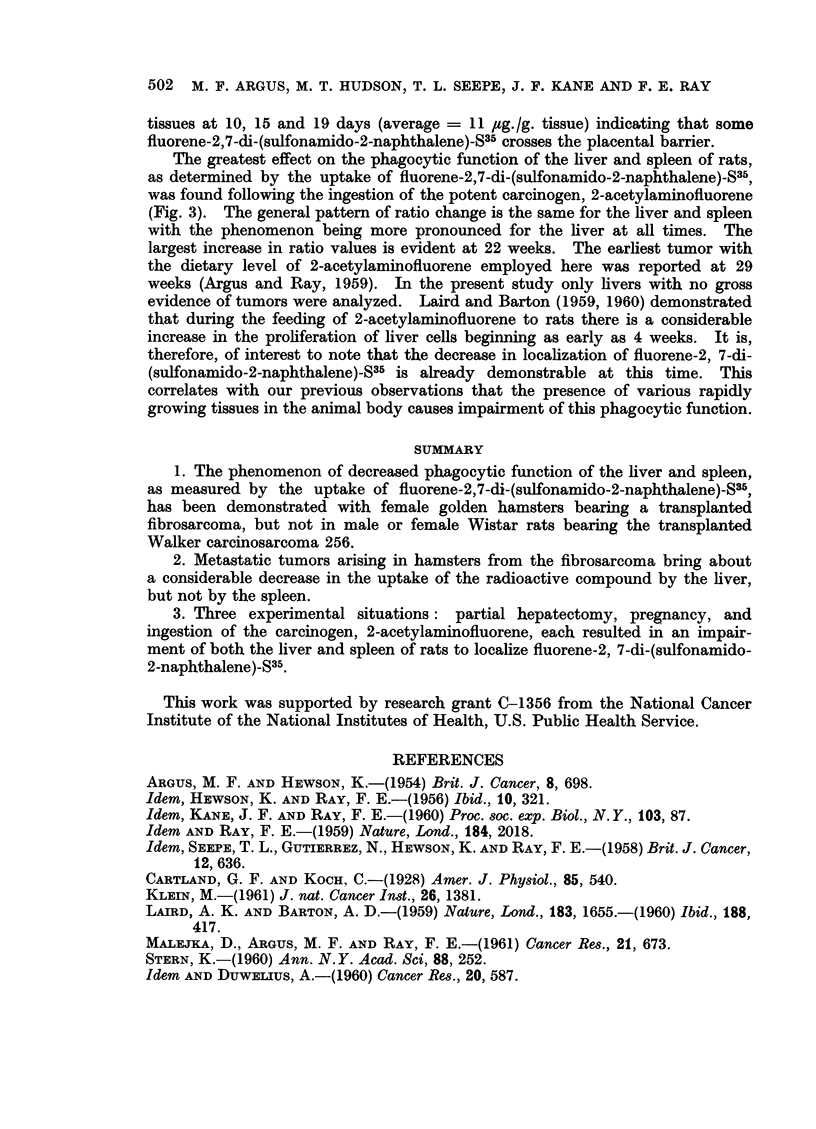# Effect of Rapid Tissue Growth on the Uptake of Fluorene-2,7-Di-(Sulfonamido-2-Naphthalene)-S^35^ by the Liver and Spleen of Rats and Hamsters

**DOI:** 10.1038/bjc.1962.57

**Published:** 1962-09

**Authors:** Mary F. Argus, Marie T. Hudson, Treva L. Seepe, Judith F. Kane, Francis E. Ray


### EFFECT OF RAPID TISSUE GROWTH ON THE UPTAKE OF FLUORENE-2,7-DI-(SULFONAMIDO-2-NAPHTHALENE)-$S^{35}$ BY THE LIVER AND SPLEEN OF RATS AND HAMSTERS

MARY F. ARGUS*, MARIE T. HUDSON, TREVA L. SEEPE, JUDITH F. KANE AND FRANCIS E. RAY

*From the Cancer Research Laboratory, University of Florida, Gainesville, Florida, U.S.A.*

Received for publication May 7, 1962

PREVIOUS studies on the localization of fluorene-2,7-di-(sulfonamido-2-naphthalene)-$S^{35}$ revealed that the presence of a subcutaneously transplanted tumor decreased the uptake of this radioactive compound by the liver and spleen of mice (Argus and Hewson, 1954 ; Argus, Hewson and Ray, 1956). This phenomenon was demonstrated in both $CAF_1$/Jax mice bearing a squamous cell stomach carcinoma and C3H mice bearing the Barrett mammary adenocarcinoma. Autoradiograms indicated that it is a phagocytic function of the reticuloendothelial system that is impaired (Argus, Hewson and Ray, 1956). Subsequent studies to determine if other stress conditions produce this phenomenon showed that, unlike the presence of a tumor, neither X-irradiation nor cortisone affects the uptake of fluorene-2,7-di-(sulfonamido-2-naphthalene)-$S^{35}$ by the liver and spleen of mice or rats (Argus, Kane and Ray, 1960 ; Malejka, Argus and Ray, 1961).

In the present studies this localization phenomenon was investigated in hamsters and rats as influenced by the presence of two different types of transplanted tumors (a fibrosarcoma and Walker carcinosarcoma 256). The effect of metastatic tumors existing after surgical removal of the transplanted sarcoma was studied in hamsters, and the effects of liver regeneration (resulting from partial hepatectomy) and of pregnancy were observed in rats. To determine if the phenomenon occurs as a result of the increase in proliferation of liver cells (Laird and Barton, 1959, 1960) caused by the carcinogen, 2-acetylaminofluorene, before the gross appearance of tumors, the uptake of fluorene-2,7-di-(sulfonamido-2-naphthalene)-$S^{35}$ by the liver and spleen of rats fed the carcinogen, 2-acetylaminofluorene, for varying periods of time was investigated.

### MATERIALS AND METHODS

Fluorene-2,7-di-(sulfonamido-2-naphthalene)-$S^{35}$ having a specific activity of 13,400 disintegrations/sec./mg. (0·36 $\mu$c/mg.) was prepared as previously reported (Argus and Hewson, 1954). For administration, the radioactive compound was dissolved (9 mg./ml.) in 0·05N NaOH except for a study of two groups of hamsters where the injection medium was dilute sodium bicarbonate and

* Present address : Departments of Medicine and Biochemistry, Tulane University Medical School, USPHS Hospital Research Laboratory, 210 State Street, New Orleans 18, Louisiana, U.S.A.

ethanol (18 mg. compound/ml.). Each rat received 12 mg. of radioactive compound *via* tail vein. Each hamster received 9 mg. of labeled compound. Administration was *via* the femoral vein except in one study where administration was intraperitoneally.

A total of 19 female golden hamsters was employed. These animals were 16 weeks old at the beginning of the experiment. Six animals received no treatment before receiving fluorene-2,7-di-(sulfonamido-2-naphthalene)-$S^{35}$ and served as controls (Groups I, III and VIII). Eleven hamsters received a subaxillary transplant of a metastasizing fibrosarcoma. This represented the thirteenth generation transplant of this tumour which originally arose spontaneously and was recently described by Klein (1961). When the tumor was 4 weeks old and had reached an average size of 3·2 g. the labeled compound was administered to 7 of these tumor-bearing hamsters (Groups II, IV and IX), while the tumor was surgically removed, under nembutal anesthesia, from 2 others (Group VI). The tumor was allowed to persist 10 weeks in 2 hamsters, at which time it had reached an average weight of 7·8 g. and was surgically removed (Group VII). Two additional animals were subjected to nembutal anesthesia and surgical manipulation similar to the tumor excision procedure (Group V). These sham operated controls, as well as those hamsters undergoing tumor excision, were allowed to recover 4 weeks before administration of the fluorene-2, 7-di-(sulfonamido-2-naphthalene)-$S^{35}$.

A total of 83 (24 male, 59 female) Wistar rats (Carworth Farms, New City, New York, U.S.A.) were used. The females for the pregnancy study were 5 to 6 months old ; all other rats were 5 to 8 weeks old at the beginning of the experiments. Seven males and 5 females received the radioactive compound without prior treatment, and these served as controls for the transplanted tumor, 2-acetylaminofluorene feeding and pregnancy studies. The Walker carcinosarcoma 256 was transplanted to the subaxillary region of 2 males and 7 females. Fluorene-2,7-di-(sulfonamido-2-naphthalene)-$S^{35}$ was administered to these rats when the tumor was 10 days old and had an average weight of 5·7 g. Fifteen male rats received, by stomach tube, 2-acetylaminofluorene suspended in 1 per cent methylcellulose. The dosage of carcinogen was 3 mg. daily, five times weekly for 20 weeks. The sulphur-35 localization study was carried out in groups of 2 or 3 animals at intervals of 4, 6, 11, 14, 22, 29 and 33 weeks following the first ingestion of 2-acetylaminofluorene. The 9 rats used in the pregnancy study were bred at the Carworth Farms and the exact date of conception was supplied with the animals. The radioactivity localization study was carried out in groups of 2 or 3 of these rats at 6, 10, 15 and 19 days after conception. At autopsy each rat used in this study had 10 to 12 fetuses. Partial hepatectomy, performed on 34 female rats, involved removal of two-thirds of the liver under nembutal anesthesia. Groups of 4 or 5 of these rats were administered the radioactive compound at 0, 6, 18, 24, 42, 66, 90, 114 and 234 hour intervals following partial hepatectomy. Four female rats, which served as the sham operated controls for the partial hepatectomy study, were subjected to nembutal anesthesia, abdominal incision and liver massage immediately before administration of fluorene-2, 7-di-(sulfonamido-2-naphthalene)-$S^{35}$.

Following adminstration of the radioactive compound, each animal was placed in an individual metabolism cage. Hamsters were killed at 8 hours and rats at 6 hours following administration of the radioactive compound. The concentration and per cent recovery of radioactive material in the tissues and excreta were determined by methods previously described (Argus, Seepe, Gutierrez, Hewson and Ray, 1958). The localization of radioactivity was determined in total fetal tissue at 10, 15 and 19 days in the pregnancy studies. Following intraperitoneal administration of fluorene-2, 7-di-(sulfonamido-2-naphthalene)-$S^{35}$ to hamsters, the radioactivity remaining in the peritoneal cavity was determined on an aliquot of fluid obtained from four washings of this cavity with 10 ml. portions of distilled water before removal of the organs. Total blood volume for rats and hamsters was calculated on the basis of 6·7 ml. blood per 100 g. body weight (Cartland and Koch, 1928).

### RESULTS AND DISCUSSION

The tissue distribution of radioactivity in hamsters following injection of fluorene-2, 7-di-(sulfonamido-2-naphthalene)-$S^{35}$ is given in Table I. Comparisons of the uptake of radioactivity by the liver and spleen of tumor-bearing and tumor-free hamsters are presented in Table II. These are expressed as ratios in the same manner as was used previously in similar studies with mice (Argus Hewson and Ray, 1956). In Groups I and II the radioactive compound was administered intravenously in a dilute bicarbonate and ethanol solution. This permitted direct comparison with the results from our previous studies with this compound in mice where the same injection medium was employed (Argus and Hewson, 1954; Argus, Hewson and Ray, 1956). The ratio values comparing Group I and Group II (Table II) show that the liver tissue of tumor-free hamsters localizes 1·85 times as much radioactivity as the liver from tumor-bearing hamsters; this ratio for the spleen is 1·80. As can be seen from the range values for Groups I and II on Table II there is no overlapping of concentration values for either organ between control and experimental animals.

Thus, the phenomenon of decreased uptake for fluorene-2, 7-di-(sulfonamido-2-naphthalene)-$S^{35}$ by the liver and spleen of tumor-bearing animals as compared to tumor-free animals was demonstrated in hamsters with a transplanted fibrosarcoma. In these and previous experiments it was difficult to maintain the radioactive compound in a uniform concentration in the injection medium. Fluorene-2, 7-di-(sulfonamido-2-naphthalene)-$S^{35}$ is soluble in 0·05 N NaOH, and the above experiment was repeated using this injection medium (Groups III and IV, Tables I and II). The ratio values were again significantly greater than 1·00, and, thus, 0·05 N NaOH was selected as the solvent for the subsequent experiments.

It was next attempted to demonstrate this phenomenon with metastatic tumors. For these studies the transplanted fibrosarcoma was completely excised at 4 weeks (Group VI) or at 10 weeks (Group VII). When the animals were killed, 4 weeks after excision, metastatic tumors of the lung and kidney were grossly evident in the hamsters of Group VII. Although metastatic tumors were not visible grossly in the animals of Group VI, Klein (1961) reported 73 per cent metastasis with this tumor following excision of the primary tumor at 4 weeks when there was no recurrence of the primary tumor and the animals were killed 13 weeks after excision of the transplanted fibrosarcoma. Sham operated hamsters (Group V) served as controls for the experimental animals of Groups VI and VII.

Comparing control Groups III and V it is evident that there is an increased localization of radioactivity in the spleen and, more pronouncedly, in the liver

TABLE I.—*Distribution of Radioactivity in Female Golden Hamsters at 8 Hours Following Intravenous or Intraperitoneal Injection of Fluorene-2, 7-Di-(Sulfonamido-2-Napthalene)-$S^{35}$*

| | Concentration in $\mu$g. compound per g. tissue, or ml. blood cells or plasma | | | | | | | | |
|---|---|---|---|---|---|---|---|---|---|
| | *Group I | Group II | Group III | Group IV | Group V | Group VI | Group VII | Group VIII | Group IX |
| Blood cells | 8 | 34 | 9 | 6 | 4 | 11 | 4 | 3 | 1 |
| Blood plasma | 18 | 52 | 12 | 9 | 19 | 22 | 25 | 11 | 5 |
| Liver | 616 | 333 | 215 | 152 | 713 | 265 | 258 | 126 | 163 |
| Lungs | 157 | 147 | 126 | 92 | 163 | 134 | 168 | 45 | 61 |
| Spleen | 117 | 65 | 38 | 20 | 65 | 49 | 67 | 195 | 57 |
| Kidney | 93 | 94 | 83 | 62 | 27 | 113 | 118 | 29 | 43 |
| Skin | 34 | 34 | 37 | 25 | 43 | 52 | 28 | 13 | 24 |
| Leg muscle | 43 | 46 | 62 | 32 | 54 | 164 | 126 | 8 | 21 |
| Stomach with contents | 57 | 133 | 32 | 42 | 45 | 108 | 60 | 70 | 30 |
| Small intestine with contents | 156 | 119 | 177 | 80 | 172 | 179 | 75 | 333 | 142 |
| Large intestine with contents | 689 | 792 | 579 | 693 | 174 | 654 | 329 | 348 | 435 |
| Carcass | 45 | 49 | 39 | 38 | 63 | 86 | 45 | 75 | 48 |
| Tumour | — | 44 | — | 57 | — | — | — | — | 15 |
| Excreta | 422 | 35 | 46 | 8 | 543 | 193 | 1458 | 28 | 34 |
| Abdominal fluid | — | — | — | — | — | — | — | 45 | 63 |
| | Percentage administered radioactivity recovered | | | | | | | | |
| | * Group I | Group II | Group III | Group IV | Group V | Group VI | Group VII | Group VIII | Group IX |
| Blood cells | 0·31 | 0·56 | 0·31 | 0·39 | 0·13 | 0·35 | 0·15 | 0·13 | 0·05 |
| Blood plasma | 0·69 | 1·89 | 0·43 | 0·42 | 0·68 | 0·74 | 0·45 | 0·45 | 0·27 |
| Liver | 20·63 | 13·40 | 8·86 | 7·99 | 26·38 | 9·92 | 10·77 | 6·83 | 9·80 |
| Lungs | 0·99 | 1·05 | 0·74 | 0·74 | 1·16 | 0·85 | 1·62 | 0·43 | 0·45 |
| Spleen | 0·14 | 0·12 | 0·05 | 0·05 | 0·10 | 0·07 | 0·12 | 0·23 | 0·18 |
| Kidney | 0·91 | 0·96 | 0·89 | 0·67 | 0·34 | 1·17 | 1·18 | 0·31 | 0·54 |
| Skin | 8·26 | 8·03 | 8·45 | 6·72 | 9·04 | 8·85 | 5·64 | 2·88 | 6·64 |
| Leg muscle | 0·25 | 0·42 | 0·57 | 0·31 | 0·34 | 1·74 | 1·09 | 0·08 | 0·37 |
| Stomach with contents | 1·53 | 0·69 | 0·85 | 0·76 | 1·03 | 2·39 | 1·25 | 1·72 | 0·83 |
| Small intestine with contents | 3·26 | 2·25 | 4·06 | 0·76 | 3·42 | 3·53 | 1·35 | 9·94 | 4·44 |
| Large intestine with contents | 40·89 | 43·70 | 35·75 | 48·09 | 8·69 | 41·48 | 21·95 | 23·81 | 36·26 |
| Carcass | 27·03 | 26·54 | 35·81 | 26·57 | 34·62 | 41·84 | 29·23 | 48·64 | 36·32 |
| Tumour | — | 2·12 | — | 2·44 | — | — | — | — | 0·72 |
| Excreta | 3·53 | 0·59 | 0·48 | 0·08 | 5·98 | 2·09 | 16·12 | 0·31 | 0·38 |
| Abdominal fluid | — | — | — | — | — | — | — | 3·79 | 3·55 |
| Total | 108·42 | 102·32 | 97·25 | 95·99 | 91·91 | 115·02 | 90·92 | 99·55 | 100·80 |

* Group I—Controls, injected i.v., compound in dilute sodium bicarbonate and ethanol, average values from 2 animals.

Group II—Bearing transplanted sarcoma 4 weeks, injected i.v., compound in dilute sodium bicarbonate and ethanol, average values from 3 animals.

Group III—Controls, injected i.v., compound in 0·05N NaOH, average values from 2 animals.

Group IV—Bearing transplanted sarcoma 4 weeks, injected i.v., compound in 0·05N NaOH, one animal.

Group V—Sham operated controls, injected i.v., compound in 0·05N NaOH, average values from 2 animals.

Group VI—Transplanted sarcoma excised at 4 weeks, injected i.v., compound in 0·05N NaOH, average values from 2 animals.

Group VII—Transplanted sarcoma excised at 10 weeks, injected i.v., compound in 0·05N NaOH, average values from 2 aminals.

Group VIII—Controls, injected i.p., compound in 0·05N NaOH, average value from 2 animals.

Group IX—Bearing transplanted sarcoma 4 weeks, injected i.p., compound in 0·05N NaOH, average values from 3 animals.

TABLE II.—*Comparative Study of the Uptake of Radioactivity by the Liver and Spleen of Female Golden Hamsters at 8 Hours Following Intravenous or Intraperitoneal Injection of Fluorene-2, 7-Di-(Sulfonamido-2-Napthalene)-$S^{35}$*

Concentration (μg. compound per g. tissue)

| | Liver | | | | | Spleen | | | | |
|---|---|---|---|---|---|---|---|---|---|---|
| | Control | | Experimental | | Ratio (Av. control / Av. experimental) | Control | | Experimental | | Ratio (Av. control / Av. experimental) |
| | Range | Average | Range | Average | | Range | Average | Range | Average | |
| *Group I | 613–618 | 616 | | | 1·85 | 83–150 | 117 | | | 1·80 |
| Group II | | | 250–375 | 333 | | | | 55–70 | 65 | |
| Group III | 202–228 | 215 | | | 1·41 | 28–48 | 38 | | | 1·90 |
| Group IV | | | 152 | 152 | | | | 20 | 20 | |
| Group V | 590–845 | 713 | | | 2·69 | 50–80 | 65 | | | 1·33 |
| Group VI | | | 232–298 | 265 | | | | 39–58 | 49 | |
| Group V | 590–845 | 713 | | | 2·76 | 50–80 | 65 | | | 0·97 |
| Group VII | | | 200–315 | 258 | | | | 63–70 | 67 | |
| Group VIII | 123–130 | 126 | | | 0·77 | 95–295 | 195 | | | 3·41 |
| Group IX | | | 140–205 | 163 | | | | 50–62 | 57 | |

* See footnote to Table I.

as a result of the sham operation (Table I). Comparing Group V to Group VI and Group VII we find that there is a definite decreased ability of the liver to localize the radioactive compound as a result of the presence of metastatic tumors (ratios 2·69 and 2·76 with no overlapping of the control and experimental range values, Table II). The effect on the spleen, however, is not significant.

The phagocytosis of colloidal gold by the liver and spleen of tumor hosts has been investigated using the intraperitoneal route of administration (*e.g.*, Stern and Duwelius, 1960). When intraperitoneal injection was employed in our studies with fluorene-2, 7-di-(sulfonamido-2-naphthalene)-$S^{35}$ we found (Table I) a decrease in the localization of radioactivity in the liver and a very marked increase in the uptake by the spleen in tumor-free hamsters receiving the compound intraperitoneally (Group VIII) as compared to tumor-free hamsters injected intravenously (Group III).

It should be pointed out that for the intraperitoneal injection no anesthesia is necessary, while the hamsters receiving fluorene-2, 7-di-(sulfonamido-2-naphthalene)-$S^{35}$ intravenously are under nembutal anesthesia, which may play a role in the noted difference in the distribution of radioactivity. When tumor-bearing hamsters receiving the intraperitoneal injection (Group IX) are compared to Group VIII (Table II), it may be seen that the presence of the transplanted sarcoma considerably decreases the ability of the spleen to localize radioactivity (ratio = 3·41), while with the liver the effect is one of increased uptake (ratio = 0·77).

The effect of transplanted Walker carcinosarcoma 256 on the distribution and comparative uptake by the liver and spleen of fluorene-2, 7-di-(sulfonamido-2-naphthalene)-$S^{35}$ in Wistar rats was studied in both male and female animals (Tables III and IV). In all cases the tissues of control female rats (Group X) localize a larger concentration of the radioactive compound than do the corresponding tissues in the control males (Group XI) This influence of sex on localiza-

TABLE III.—*Distribution of Radioactivity at 6 Hours Following Intravenous Injection of Fluorene-2,7-Di(Sulfonamido-2-Naphthalene)-$S^{35}$ To Wistar Rats With and Without a Transplanted Walker Carcinosarcoma 256*

| | Concentration in μg. compound per g. tissue, or ml. blood cells or plasma | | | | Percentage administered radioactivity recovered | | | |
|---|---|---|---|---|---|---|---|---|
| | * Group X | Group XI | Group XII | Group XIII | Group X | Group XI | Group XII | Group XIII |
| Blood cells . . . . | 4 | 7 | 1 | 5 | 0·15 | 0·32 | 0·06 | 0·17 |
| Blood plasma . . . | 8 | 8 | 6 | 9 | 0·32 | 0·35 | 0·35 | 0·35 |
| Liver . . . . | 176 | 142 | 80 | 79 | 8·51 | 9·37 | 5·11 | 4·51 |
| Lungs . . . . | 168 | 107 | 74 | 210 | 1·14 | 1·79 | 2·46 | 0·59 |
| Spleen . . . . | 52 | 50 | 21 | 20 | 0·17 | 0·50 | 0·11 | 0·30 |
| Kidney . . . . | 82 | 56 | 35 | 43 | 0·84 | 0·60 | 0·42 | 0·49 |
| Thymus . . . . | 50 | 41 | 22 | 27 | 0·12 | 0·10 | 0·07 | 0·09 |
| Skin . . . . . | 78 | 91 | 40 | 125 | 17·54 | 23·12 | 19·77 | 29·01 |
| Leg muscle . . . | 45 | 34 | 23 | 42 | 0·49 | 0·35 | 0·26 | 0·31 |
| Stomach with contents . | 48 | 23 | 30 | 13 | 0·53 | 0·37 | 0·47 | 0·23 |
| Small intestine with contents | 398 | 289 | 174 | 270 | 17·33 | 14·98 | 9·30 | 14·65 |
| Large intestine with contents | 373 | 346 | 896 | 835 | 22·33 | 20·98 | 52·74 | 36·79 |
| Carcass . . . . | 51 | 38 | 21 | 45 | 31·27 | 22·20 | 22·62 | 21·02 |
| Tumor . . . . | — | 41 | — | 61 | — | 3·20 | — | 3·35 |
| Excreta . . . . | 16 | 19 | 42 | 972 | 0·11 | 0·15 | 1·33 | 5·91 |
| Total . | | | | | 100·85 | 98·38 | 115·07 | 117·77 |

* Group X—Female controls, average values from 5 animals.
Group XI—Female tumor-bearing, average values from 7 animals.
Group XII—Male contols, average values from 7 animals
Group XIII—Male tumor-bearing, average values from 2 animals.

TABLE IV.—*Comparative Study of the Uptake of Radioactivity by the Liver and Spleen of Wistar Rats at 6 Hours Following Intravenous Injection of Fluorene-2, 7-Di-(Sulfonamido-2-Naphthalene)-$S^{35}$*

| | Concentration (μg. compound per g. tissue) | | | | | | | | | |
|---|---|---|---|---|---|---|---|---|---|---|
| | Liver | | | | | Spleen | | | | |
| | Control | | Experimental | | Ratio (Av. control / Av. experimental) | Control | | Experimental | | Ratio (Av. control / Av. experimental) |
| * | Range | Average | Range | Average | | Range | Average | Range | Average | |
| Group X | 148–230 | 176 | | | 1·24 | 45–60 | 52 | | | 1·04 |
| Group XI | | | 78–190 | 142 | | | | 33–60 | 50 | |
| Group XII | 52–175 | 80 | | | 1·01 | 13–57 | 21 | | | 1·05 |
| Group XIII | | | 70–87 | 79 | | | | 17–33 | 20 | |

* See footnote to Table III.

tion has also been reported in other studies with this compound in rats (Argus, Kane and Ray, 1960; Malejka, Argus and Ray, 1961). As evidenced by the overlapping of range values and the ratios not significantly different from unity (Table IV), the phenomenon of decreased localization in the liver and spleen, observed in tumor-bearing mice and hamsters, is not manifest in either sex of this strain of rat with this transplanted tumor.

The influence of the regeneration of liver tissue resulting from partial hepatectomy does, however, show a definite effect on the phagocytic function of the liver and spleen (Fig. 1). Up to 240 hours following the operation, both these organs show a reduced ability to localize fluorene-2,7-di-(sulfonamido-2-naphthalene)-$S^{35}$ since the ratios are always greater than 1·00. The liver and spleen follow the

FIG. 1.—Effect of partial hepatectomy on the uptake of fluorene-2,7-di-(sulfonamido-2-naphthalene)-$S^{35}$ by the liver and spleen of rats expressed as the ratio : average concentration of the sham operated controls over the average concentration of the experimentals. The ratios are plotted as a function of the time in hours following partial hepatectomy.

same pattern of ratio change with time. This apparent decrease in the phagocytic function of the reticuloendothelial system of these organs as a result of active liver regeneration is in opposition to the data reported by Stern (1960). He reports an increase in the per cent of injected colloidal gold (per gram of tissue) phagocytized by the liver of rats at 3, 8 and 16 days following partial hepatectomy. In these studies $Au^{198}$ was administered by intracardiac injection 24 hours before the partial hepatectomy, and the localization values obtained were compared to controls undergoing the sham operation at the same time interval before killing. In our studies, facilities would not permit the sham operation of groups of animals at each of the time intervals. Thus, all experimental (partially hepatectomized) rats are compared to the same sham operated controls killed at 6 hours. As reported for the hamsters, sham operation increases the uptake of radioactivity by the liver and spleen when compared to the values for these organs from untreated controls. For this reason the ratio values in Fig. 1 at the later time intervals may be exaggerated since the experimental rats would have had more time to recover from the effects of the operative procedures than the controls. However, even if the values for the experimentals are compared to non-operated control values, the ratios are still greater than 1·00 and indicate consequently a decreased phagocytic function rather than the

FIG. 2.—Influence of pregnancy on the uptake of fluorene-2,7-di-(sulfonamido-2-naphthalene) $S^{35}$ by the liver and spleen of rats expressed as the ratio : average concentration of the untreated controls over the average concentration of the experimentals. The ratios are plotted as a function of the days from conception.

FIG. 3.—Effect of the ingestion of 2-acetylaminofluorene on the uptake of fluorene-2,7-di-(sulfonamido-2-naphthalene)-$S^{35}$ by the liver and spleen of rats expressed as the ratio : average concentration of the untreated controls over the average concentration of the experimentals. The ratios are plotted as a function of the time in weeks from the first ingestion of the carcinogen. Feeding of 2-acetylaminofluorene was maintained up to the 20th week.

increased phagocytosis reported by Stern (1960) under his experimental conditions.

That the presence of developing fetuses also influences the uptake of fluorene-2, 7-di-(sulfonamido-2-napthalene)-$S^{35}$ by the liver and spleen is shown in Fig. 2. The values for the spleen indicate a maximum depression in phagocytosis between 10 and 19 days following conception, while for the liver there is a gradual increase in the ratio values with time. Low levels of radioactivity were found in the fetal tissues at 10, 15 and 19 days (average = 11 $\mu$g./g. tissue) indicating that some fluorene-2,7-di-(sulfonamido-2-naphthalene)-$S^{35}$ crosses the placental barrier.

The greatest effect on the phagocytic function of the liver and spleen of rats, as determined by the uptake of fluorene-2,7-di-(sulfonamido-2-naphthalene)-$S^{35}$, was found following the ingestion of the potent carcinogen, 2-acetylaminofluorene (Fig. 3). The general pattern of ratio change is the same for the liver and spleen with the phenomenon being more pronounced for the liver at all times. The largest increase in ratio values is evident at 22 weeks. The earliest tumor with the dietary level of 2-acetylaminofluorene employed here was reported at 29 weeks (Argus and Ray, 1959). In the present study only livers with no gross evidence of tumors were analyzed. Laird and Barton (1959, 1960) demonstrated that during the feeding of 2-acetylaminofluorene to rats there is a considerable increase in the proliferation of liver cells beginning as early as 4 weeks. It is, therefore, of interest to note that the decrease in localization of fluorene-2, 7-di-(sulfonamido-2-naphthalene)-$S^{35}$ is already demonstrable at this time. This correlates with our previous observations that the presence of various rapidly growing tissues in the animal body causes impairment of this phagocytic function.

### SUMMARY

1. The phenomenon of decreased phagocytic function of the liver and spleen, as measured by the uptake of fluorene-2,7-di-(sulfonamido-2-naphthalene)-$S^{35}$, has been demonstrated with female golden hamsters bearing a transplanted fibrosarcoma, but not in male or female Wistar rats bearing the transplanted Walker carcinosarcoma 256.

2. Metastatic tumors arising in hamsters from the fibrosarcoma bring about a considerable decrease in the uptake of the radioactive compound by the liver, but not by the spleen.

3. Three experimental situations: partial hepatectomy, pregnancy, and ingestion of the carcinogen, 2-acetylaminofluorene, each resulted in an impairment of both the liver and spleen of rats to localize fluorene-2, 7-di-(sulfonamido-2-naphthalene)-$S^{35}$.

This work was supported by research grant C–1356 from the National Cancer Institute of the National Institutes of Health, U.S. Public Health Service.

### REFERENCES

Argus, M. F. and Hewson, K.—(1954) *Brit. J. Cancer*, **8**, 698.
*Idem*, Hewson, K. and Ray, F. E.—(1956) *Ibid.*, **10**, 321.
*Idem*, Kane, J. F. and Ray, F. E.—(1960) *Proc. soc. exp. Biol., N.Y.*, **103**, 87.
*Idem* and Ray, F. E.—(1959) *Nature, Lond.*, **184**, 2018.
*Idem*, Seepe, T. L., Gutierrez, N., Hewson, K. and Ray, F. E.—(1958) *Brit. J. Cancer*, **12**, 636.
Cartland, G. F. and Koch, C.—(1928) *Amer. J. Physiol.*, **85**, 540.
Klein, M.—(1961) *J. nat. Cancer Inst.*, **26**, 1381.
Laird, A. K. and Barton, A. D.—(1959) *Nature, Lond.*, **183**, 1655.—(1960) *Ibid.*, **188**, 417.
Malejka, D., Argus, M. F. and Ray, F. E.—(1961) *Cancer Res.*, **21**, 673.
Stern, K.—(1960) *Ann. N.Y. Acad. Sci*, **88**, 252.
*Idem* and Duwelius, A.—(1960) *Cancer Res.*, **20**, 587.