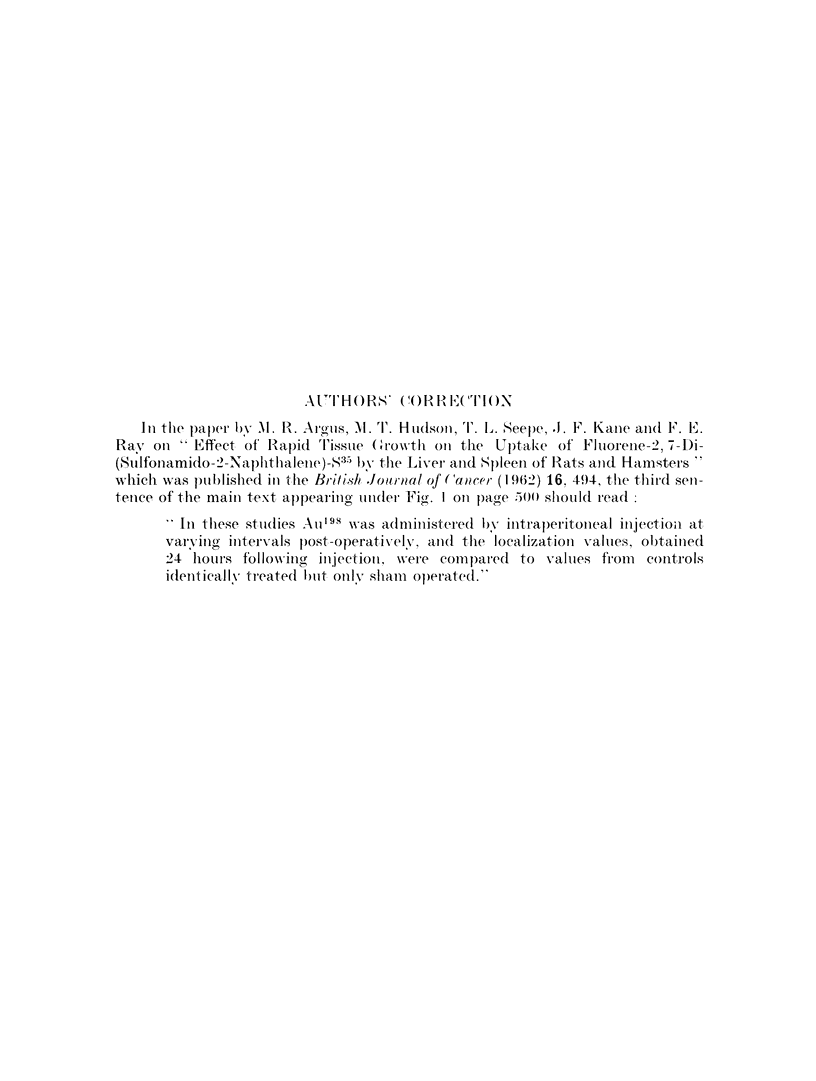# Authors' Correction

**Published:** 1963-03

**Authors:** 


### AUTHORS' CORRECTION

In the paper by M. R. Argus, M. T. Hudson, T. L. Seepe, J. F. Kane and F. E. Ray on " Effect of Rapid Tissue Growth on the Uptake of Fluorene-2,7-Di-(Sulfonamido-2-Naphthalene)-$S^{35}$ by the Liver and Spleen of Rats and Hamsters " which was published in the *British Journal of Cancer* (1962) **16**, 494, the third sentence of the main text appearing under Fig. 1 on page 500 should read :

> " In these studies $Au^{198}$ was administered by intraperitoneal injection at varying intervals post-operatively, and the localization values, obtained 24 hours following injection, were compared to values from controls identically treated but only sham operated."